# Critical roles of *Rickettsia parkeri* outer membrane protein B (OmpB) in the tick host

**DOI:** 10.1128/iai.00515-23

**Published:** 2024-01-11

**Authors:** Natthida Tongluan, Patrik Engström, Krit Jirakanwisal, Ingeborg M. Langohr, Matthew D. Welch, Kevin R. Macaluso

**Affiliations:** 1 Department of Microbiology and Immunology, University of South Alabama, Frederick P. Whiddon College of Medicine, Mobile, Alabama, USA; 2 Department of Molecular and Cell Biology, University of California, Berkeley, California, USA; 3 Department of Pathobiological Sciences, Louisiana State University, School of Veterinary Medicine, Baton Rouge, Louisiana, USA; Washington State University, Pullman, Washington, USA

**Keywords:** *Rickettsia parkeri*, outer membrane protein B, *Amblyomma maculatum*, dissemination, infection

## Abstract

*Rickettsia parkeri* is a pathogen of public health concern and transmitted by the Gulf Coast tick, *Amblyomma maculatum*. Rickettsiae are obligate intracellular bacteria that enter and replicate in diverse host cells. Rickettsial outer membrane protein B (OmpB) functions in bacterial adhesion, invasion, and avoidance of cell-autonomous immunity in mammalian cell infection, but the function of OmpB in arthropod infection is unknown. In this study, the function of *R. parkeri* OmpB was evaluated in the tick host. *R. parkeri* wild-type and *R. parkeri ompB^STOP^::tn* (non-functional OmpB) were capillary fed to naïve *A. maculatum* ticks to investigate dissemination in the tick and transmission to vertebrates. Ticks exposed to *R. parkeri* wild-type had greater rickettsial loads in all organs than ticks exposed to *R. parkeri ompB^STOP^::tn* at 12 h post-capillary feeding and after 1 day of feeding on host. In rats that were exposed to *R. parkeri ompB^STOP^::tn*-infected ticks, dermal inflammation at the bite site was less compared to *R. parkeri* wild-type-infected ticks. *In vitro*, *R. parkeri ompB^STOP^::tn* cell attachment to tick cells was reduced, and host cell invasion of the mutant was initially reduced but eventually returned to the level of *R. parkeri* wild-type by 90 min post-infection. *R. parkeri ompB^STOP^::tn* and *R. parkeri* wild-type had similar growth kinetics in the tick cells, suggesting that OmpB is not essential for *R. parkeri* replication in tick cells. These results indicate that *R. parkeri* OmpB functions in rickettsial attachment and internalization to tick cells and pathogenicity during tick infection.

## INTRODUCTION

Tick-borne rickettsial diseases, caused by obligate intracellular bacteria primarily in the genus *Rickettsia*, account for one-fourth of all tick-borne diseases in the United States (1, 2). Human infections caused by tick-borne rickettsial pathogens within the spotted fever group *Rickettsia* species present as acute febrile illnesses with fever, headache, malaise, myalgia, rash, and occasional necrotic eschars at the site of the tick bite. The spotted fever group *Rickettsia* species, *Rickettsia parkeri,* is an agent of human rickettsiosis throughout the Americas (3, 4). In the United States, *R. parkeri* is transmitted vertically between tick life cycle stages and horizontally from tick to vertebrate host by its vector, *Amblyomma maculatum*, which has diverse hosts and is expanding in geographic distribution (5, 6). Despite the emergence of *R. parkeri* and increasing range of *A. maculatum*, little is known about molecular interactions between the pathogen and its vector which is key to developing control strategies.

Rickettsial factors essential to intracellular viability have been identified for vertebrate hosts. During infection of mammalian cells, several rickettsial molecules including surface cell antigen 0 [Sca0, also known as outer membrane protein A (OmpA)], Sca1, Sca2, Sca4, and Sca5 (also known as OmpB) function in adhesion, host cell entry, and dissemination (7–11). In addition to facilitating rickettsial colonization in mice, OmpB promotes autophagy avoidance in macrophages (12, 13). However, limited information is available about OmpB function in the tick vector.

During tick feeding, ingested rickettsiae must disseminate out of the gut to the salivary glands for horizontal transmission to a subsequent vertebrate host (14–16). Tick-borne bacterial pathogens express host- and tissue-specific virulence factors (17, 18). For *R. parkeri*, proteins responsible for actin-based motility, RickA and Sca2, are functional in the tick host but not essential for dissemination to tick tissues (19). In addition, transcriptomic analysis of *Rickettsia rickettsii* in *Amblyomma* ticks revealed increased expression of *ompB* with temperature elevation during blood feeding (20, 21). Although a distinct rickettsial determinant for tick infection has not been identified, the upregulated expression of *ompB* during tick feeding suggests that OmpB may function in tick infection, dissemination, and transmission. Thus, we hypothesized that if OmpB is essential for infection of the tick, then disruption of *R. parkeri ompB* may influence infection or transmission.

The purpose of the present study was to evaluate the function of *R. parkeri* OmpB in tick cells and in the tick vector. The *R. parkeri ompB^STOP^::tn* mutant (12), which is a strain without functional OmpB production was used. *R. parkeri* wild-type (strain Portsmouth) and *R. parkeri ompB^STOP^::tn* were inoculated into tick-derived ISE6 and mammalian-derived Vero cell cultures and evaluated for cell association, internalization, and growth kinetics. Furthermore, both *R. parkeri* strains were assessed *in vivo* for tick infection, dissemination, and transmission. Distinct infection phenotypes were observed between tick- and mammalian-derived cells. The data suggest that OmpB plays a role in early attachment/infection in both tick cells and tick vector, but these differences are mitigated over time. While *R. parkeri* OmpB appears to be dispensable *in vitro*, it contributes to rickettsial infection and dissemination in the tick vector.

## RESULTS

### OmpB is important for rickettsial association with tick-derived cells

**Fig 1:**
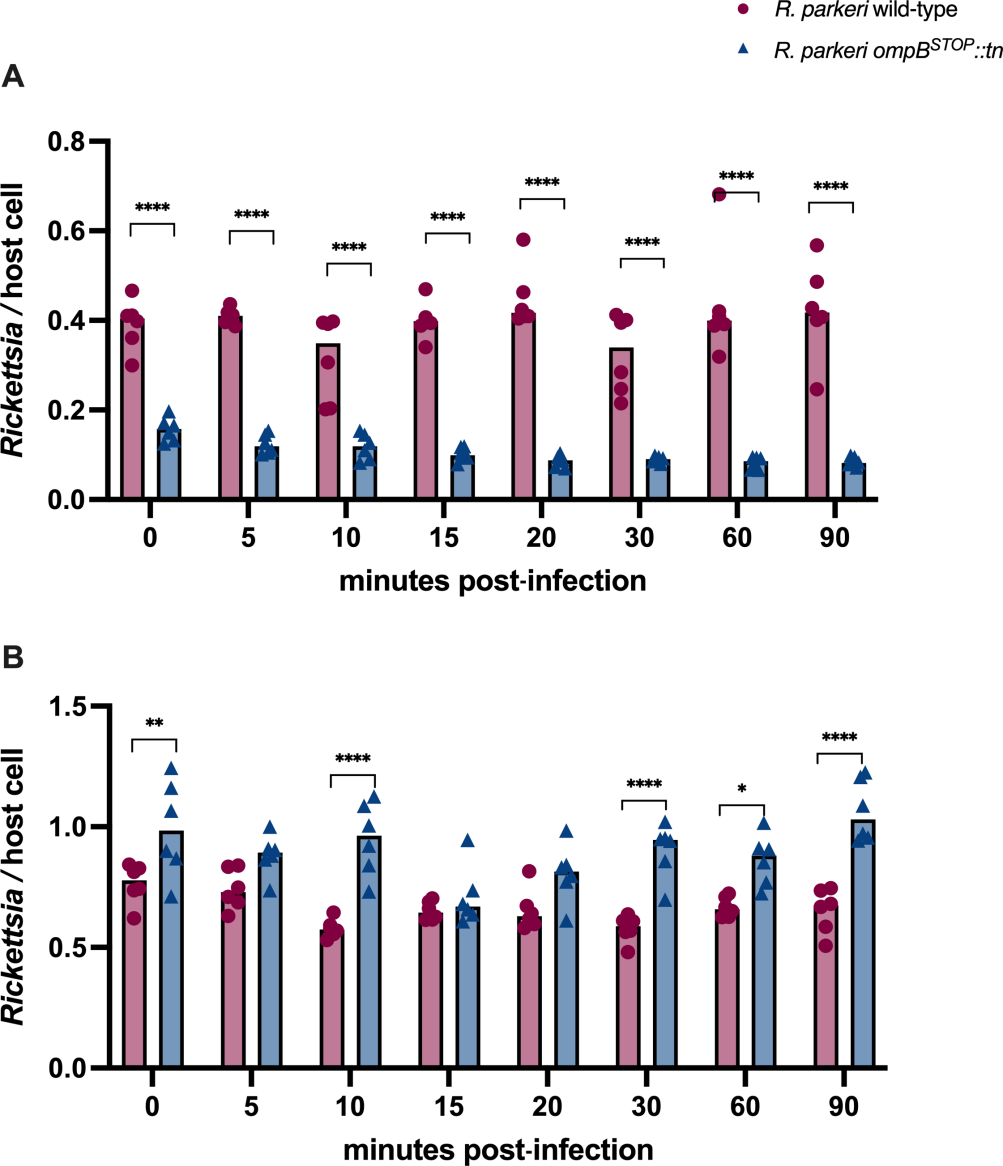
Cell association of *R. parkeri* wild-type and *R. parkeri ompB^STOP^::tn*. (**A**) Tick-derived ISE6 cells. (**B**) Vero cells. Rickettsiae were inoculated onto cells (multiplicity of infection, 5). After cells and supernatant were collected at different times, rickettsiae were quantified by qPCR. Each bar represents six wells for two biological replicates (three wells/replicate). Statistical analysis with two-way ANOVA and Šidák multiple comparison test. *P < 0.05; **P < 0.01; ****P < 0.0001.

Rickettsial OmpB is required for cell adhesion, invasion, and avoidance of autophagy in vertebrate host cells (10, 12). In this study, a mutant strain of *R. parkeri* (*R. parkeri ompB^STOP^::tn*) was used that carries a transposon insertion in *ompB*. This insertion results in a premature stop codon and renders the OmpB protein non-functional (12). The clonality of *R. parkeri ompB^STOP^::tn* was confirmed by PCR and Western blot (Fig. S1). To investigate the role of OmpB in *R. parkeri* association with ISE6 cells, cells were infected with *R. parkeri* wild-type or *R. parkeri ompB^STOP^::tn* at a multiplicity of infection (MOI) of 5. Cells were collected at 0, 5, 10, 15, 20, 30, 60, and 90 min post-infection (mpi), and *Rickettsia* and host cell genes were enumerated by qPCR. In ISE6 cells, *R. parkeri ompB^STOP^::tn* had 60%–82% less association (amount of rickettsiae per host cells) than *R. parkeri* wild-type from 0 to 90 min after infection (Fig. 1A). In Vero cells, an 8%–40% increase in association of *R. parkeri ompB^STOP^::tn* was significantly higher in most time points compared to *R. parkeri* wild-type (Fig. 1B). Therefore, OmpB may facilitate adherence to ISE6 cells but not Vero cells.

**Fig 2:**
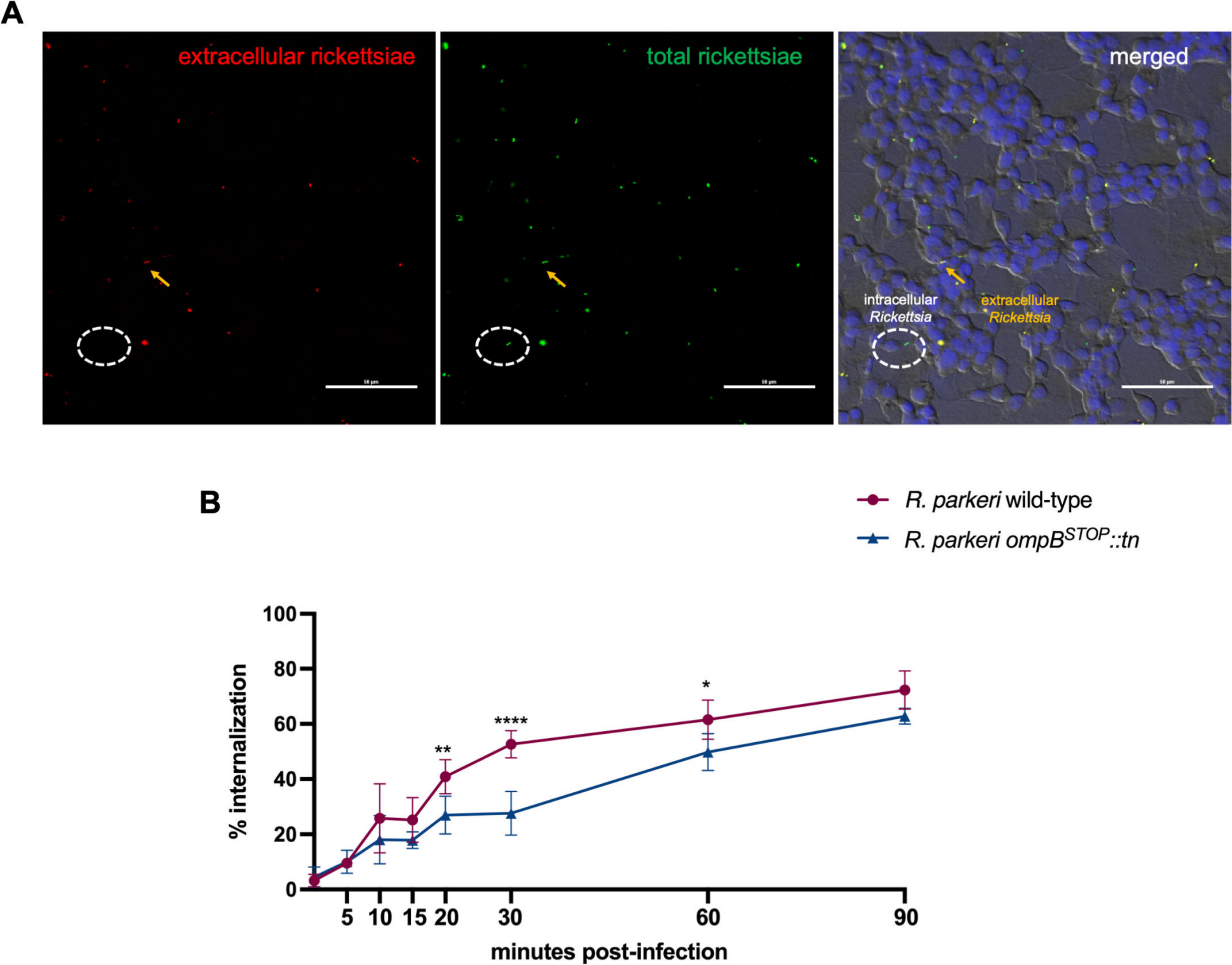
Internalization of *R. parkeri* wild-type and *R. parkeri ompB^STOP^::tn* in ISE6 cells. (**A**) Immunofluorescence of ISE6 cells infected with rickettsiae (MOI 5). Left panel: extracellular rickettsiae only are labeled with Alexa 594 (red). Middle panel: after permeabilization, all rickettsiae are labeled with Alexa 488 (green). Right panel: intracellular rickettsiae (green) determined by signal subtraction (all minus extracellular rickettsiae) (yellow). Dashed oval, representative intracellular rickettsiae. Yellow arrow, representative extracellular rickettsiae. Scale bar, 50 µm. (**B**) Internalization of rickettsiae in ISE6 cells after infection, reported as percentage of intracellular to total rickettsiae. Points represent 20 fields for 2 independent replicates (10 fields/replicate). Comparisons between *R. parkeri* wild-type and *R. parkeri ompB^STOP^::tn* with two-way ANOVA and Šidák multiple comparison test. *P < 0.05; **P < 0.01; ****P < 0.0001.

**Fig 3:**
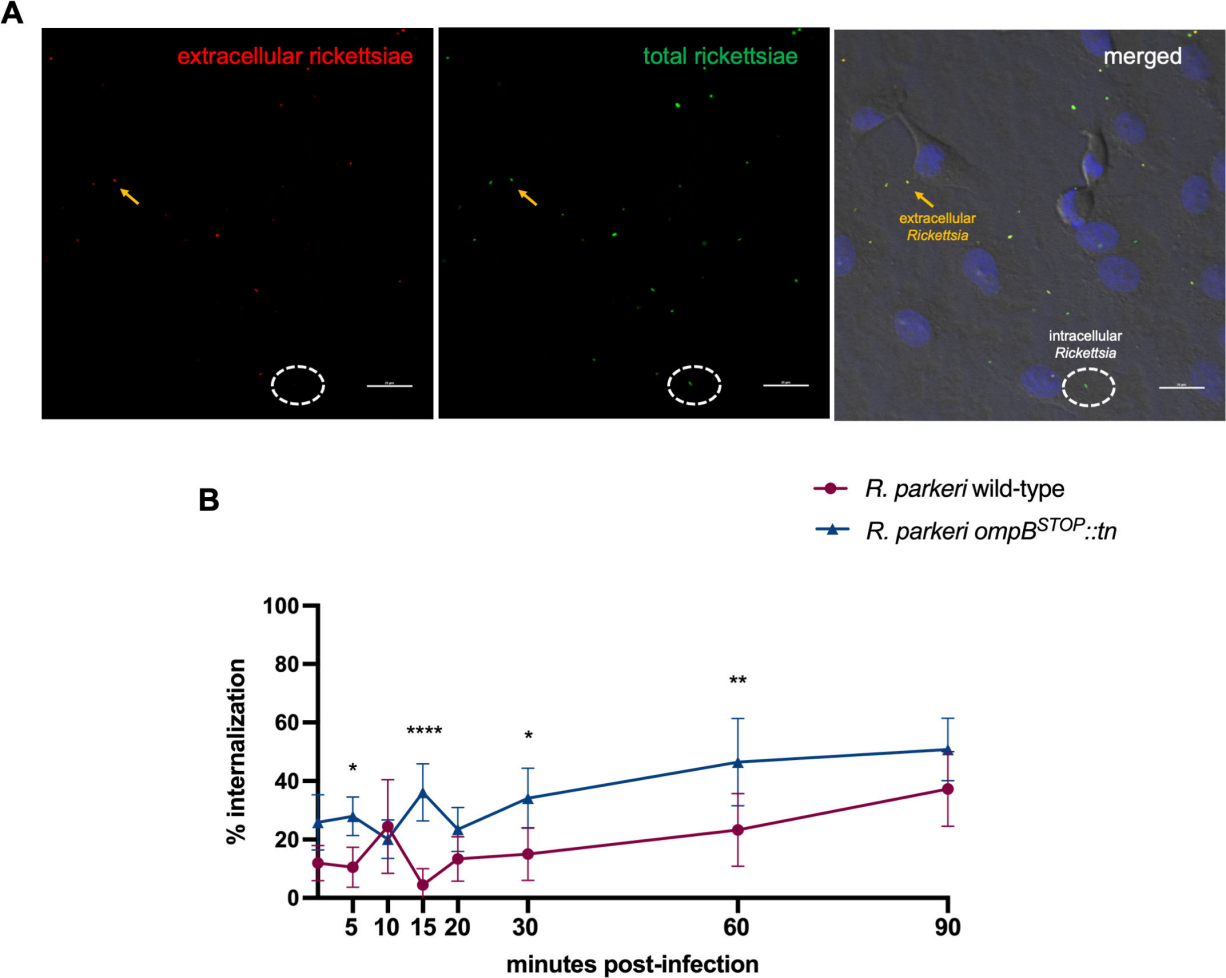
Internalization of *R. parkeri* wild-type and *R. parkeri ompB^STOP^::tn* in Vero cells. (**A**) Immunofluorescence of Vero cells infected with rickettsiae (multiplicity of infection, 5). Left panel: extracellular rickettsiae only are labeled with Alexa 594 (red). Middle panel: after permeabilization, all rickettsiae are labeled with Alexa 488 (green). Right panel: the intracellular rickettsiae were determined by signal subtraction (total rickettsiae minus extracellular rickettsiae). Dashed oval, representative intracellular rickettsiae. Yellow arrow, representative extracellular rickettsiae. Scale bar, 50 µm. (**B**) Internalization of rickettsiae in Vero cells after infection, reported as percentage of intracellular to total rickettsiae. Points represent 20 fields for 2 independent replicates (10 fields/replicate). Comparisons between *R. parkeri* wild-type and *R. parkeri ompB^STOP^::tn* with two-way ANOVA and Šidák multiple comparison test. *P < 0.05; **P < 0.01; ****P < 0.0001.

The qPCR assay was limited because it quantifies both rickettsiae that adhered to and those that invade host cells; thus, IFA was used to differentiate between adherent and intracellular bacteria (Fig. 2A) (12, 22, 23). In ISE6 cells at 20, 30, and 60 mpi, there were significant decreases in internalization of *R. parkeri ompB^STOP^::tn*, yet no significant differences in internalization between *R. parkeri* wild-type and *R. parkeri ompB^STOP^::tn* were observed by 90 mpi (Fig. 2B). The same assay was used to distinguish adherent and intracellular rickettsiae in Vero cells (Fig. 3A) and revealed that *R. parkeri ompB^STOP^::tn* was able to invade epithelial cells significantly better than *R. parkeri* wild-type up to 60 mpi, with no significant difference in the ability of either rickettsial strain to enter Vero cells at 90 mpi (Fig. 3B). The data suggest OmpB may be required for rickettsial internalization in ISE6 cells up to 60 min after infection.

### *R. parkeri ompB^STOP^::tn* display similar growth kinetics as the wild- type in ISE6 cells

**Fig 4:**
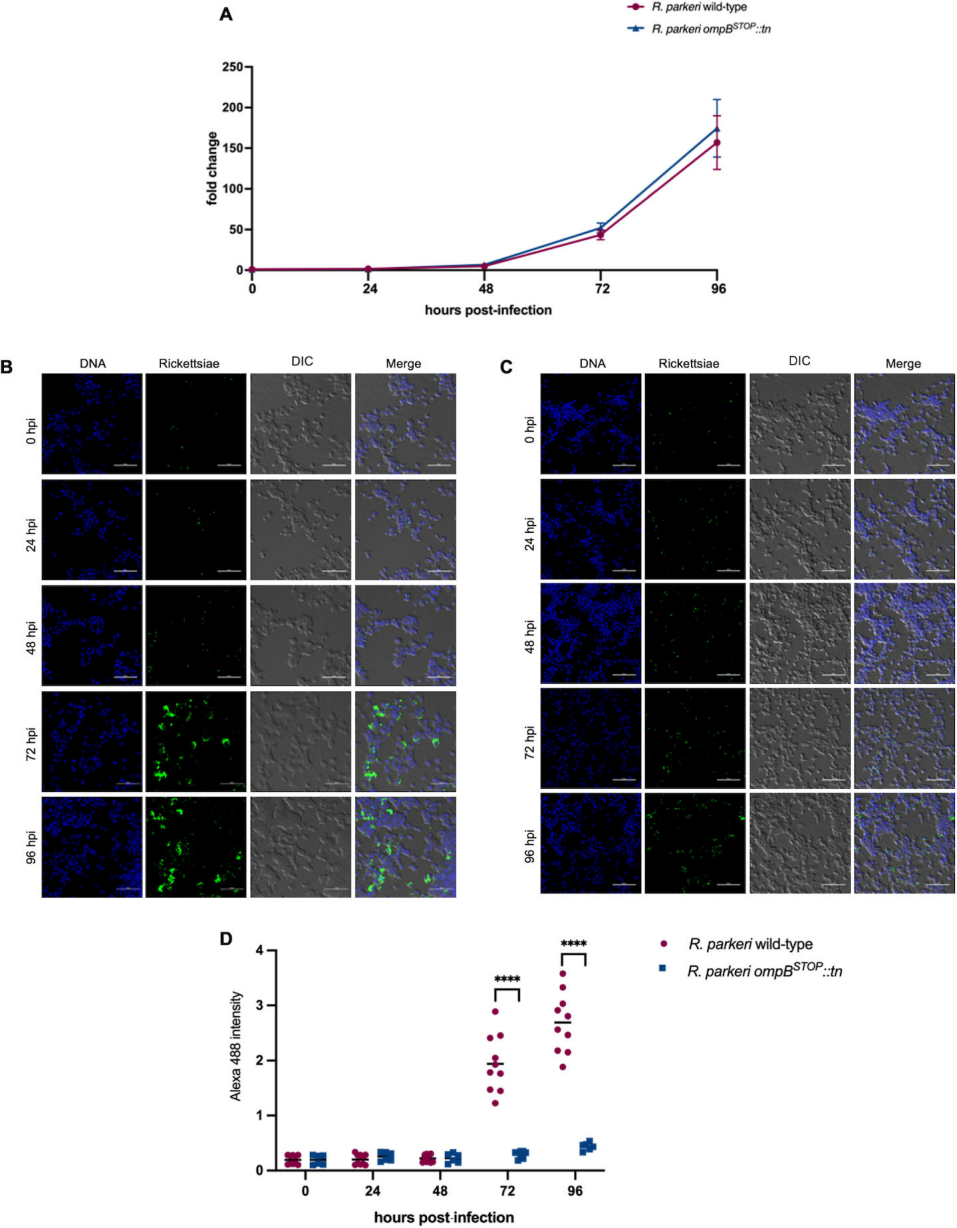
Growth kinetics and immunofluorescence of *R. parkeri* wild-type and *R. parkeri ompB^STOP^::tn* in ISE6 cells. (**A**) Infected tick cells were processed for qPCR. (**B**) Microscopy images of *R. parkeri* wild-type-infected ISE6 cells at 0, 24, 48, 72, and 96 hpi. Data represent six wells for two independent replicates (three wells/replicate). Scale bar = 50 µm. (**C**) Microscopy images of *R. parkeri ompB^STOP^::tn*-infected ISE6 cells at 0, 24, 48, 72, and 96 hpi. Data represent six wells for two independent replicates (three wells/replicate). Scale bar = 50 µm. (**D**) The intensity of Alexa 488 was measured using ImageJ from five fields per replicate. Statistical comparisons of two independent replicates (five fields per replicate) between *R. parkeri* wild-type and *R. parkeri ompB^STOP^::tn* with two-way ANOVA and Šidák multiple comparison test. ****P < 0.0001.

OmpB is dispensable for rickettsial growth in human dermal microvascular endothelial cells but essential for *R. parkeri* growth in macrophages (12). To assess potential differences in the growth kinetics between *R. parkeri* wild-type and *R. parkeri ompB^STOP^::tn* in ISE6 and Vero cells, rickettsial infections (MOI 1) were performed as described previously (24). The growth kinetics were indistinguishable between *R. parkeri* wild-type and *R. parkeri ompB^STOP^::tn* in ISE6 cells (Fig. 4A), similar to previous observations of *R. parkeri* mutants in ISE6 cells (19). However, IFA showed differences in the infection phenotypes between *R. parkeri* wild-type and *R. parkeri ompB^STOP^::tn* in ISE6 cells. Specifically, a large amount of *R. parkeri* wild-type per cell was observed at 72 and 96 h after infection (Fig. 4B), whereas fewer *R. parkeri ompB^STOP^::tn* per cell were observed in ISE6 cells (Fig. 4C). The differences observed between the results of qPCR and IFA were due to the inclusion of extracellular rickettsiae in qPCR (Fig. 4D). Therefore, *R. parkeri* wild-type and *R. parkeri ompB^STOP^::tn* had similar growth kinetics but different dissemination patterns in ISE6 cells.

**Fig 5:**
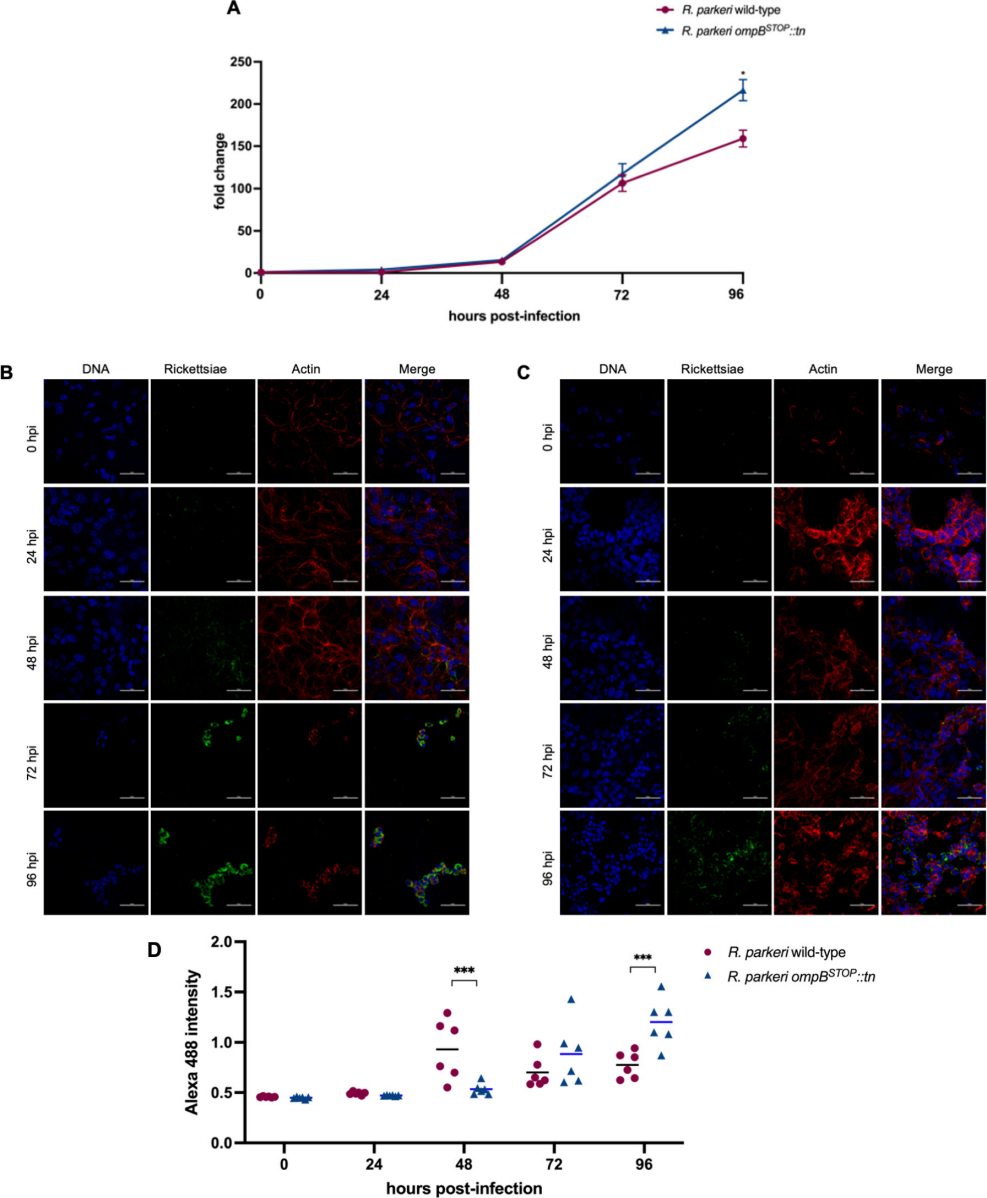
Growth kinetics and immunofluorescence of *R. parkeri* wild-type and *R. parkeri ompB^STOP^::tn* in Vero cells. (**A**) Infected Vero cells were processed for qPCR. Statistical comparisons between *R. parkeri* wild-type and *R. parkeri ompB^STOP^::tn* with two-way ANOVA. *P < 0.05. (**B**) Microscopy images of *R. parkeri* wild-type-infected Vero cells at 0, 24, 48, 72, and 96 hpi. Data represent six wells for two independent replicates (three wells/replicate). Scale bar = 50 µm. (**C**) Microscopy images of *R. parkeri ompB^STOP^::tn*-infected Vero cells at 0, 24, 48, 72, and 96 hpi. Data represent six wells for two independent replicates (three wells/replicate). Scale bar = 50 µm. (**D**) The intensity of Alexa 488 was measured using ImageJ from three fields per replicate. Statistical comparisons of two independent replicates between *R. parkeri* wild-type and *R. parkeri ompB^STOP^::tn* with two-way ANOVA and Šidák multiple comparison test. ***P < 0.001.

In Vero cells, the growth kinetics between *R. parkeri* wild-type and *R. parkeri ompB^STOP^::tn* were indistinguishable from 0 to 72 h after infection, but there was more *R. parkeri ompB^STOP^::tn* than *R. parkeri* wild-type at 96 h after infection (Fig. 5A). IFA showed that most cells were infected by both strains from 0 to 48 h after infection. From 72 to 96 h after *R. parkeri* wild-type infection, the host cell monolayers were detached (Fig. 5B); in contrast, host cells infected with *R. parkeri ompB^STOP^::tn* were evenly distributed throughout the monolayer without cell detachment (Fig. 5C). The differences in growth kinetics of Vero cells at 96 h after infection with *R. parkeri* wild-type versus *R. parkeri ompB^STOP^::tn* may be attributed to the observed differences in monolayer integrity.

### Reduced rickettsial loads in *A. maculatum* tick organs after exposure to *R. parkeri ompB^STOP^::tn* versus *R. parkeri* wild-type

**Fig 6:**
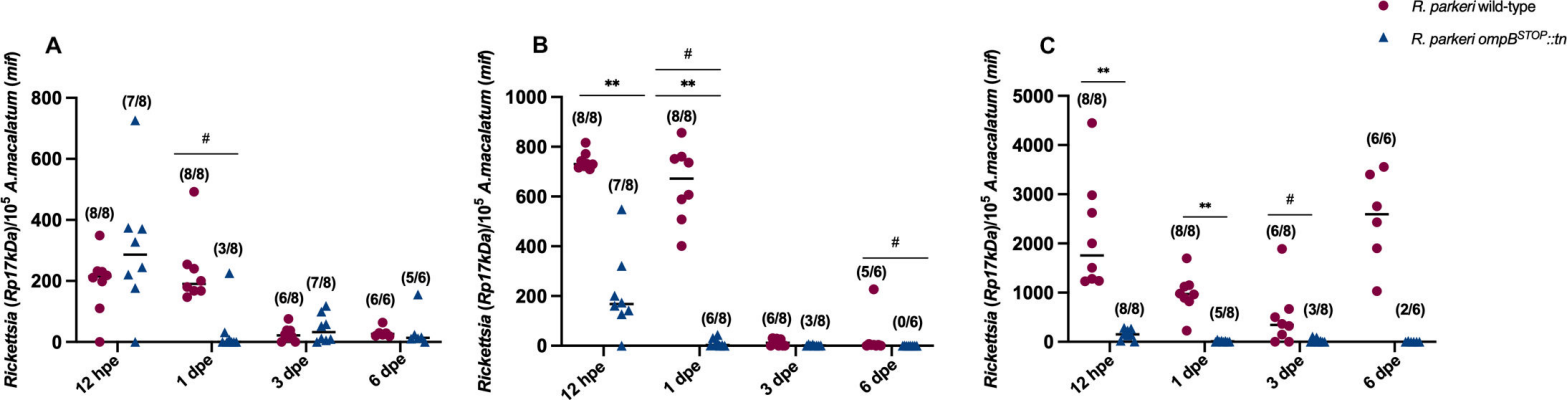
Rickettsial loads and prevalence in *A. maculatum* tissues. The numbers of rickettsiae (17 kDa) per 10^5^ tick cells were determined at various times after infection in tick (**A**) salivary glands, (**B**) midguts, and (**C**) ovaries. Values in parentheses are the proportion of ticks that were infected/total number of ticks. Statistical comparisons of two independent replicates between *R. parkeri* wild-type and *R. parkeri ompB^STOP^::tn* with two-way ANOVA for rickettsial load (**P < 0.001) and Fisher exact test for rickettsial prevalence (#*P* < 0.05).

The *in vitro* results showed that loss of OmpB impaired rickettsial attachment and internalization in ISE6 cells, but OmpB was dispensable for replication in ISE6 cells. To determine rickettsial dissemination in ticks, infected ticks were assessed after exposure to rickettsiae by capillary feeding (12 h) and after being returned to the host for 1, 3, and 6 days. Tick salivary glands, midguts, and ovaries were recovered and assessed individually for rickettsial prevalence and load by qPCR. *R. parkeri* wild-type and *R. parkeri ompB^STOP^::tn* infection showed varied rickettsial dissemination throughout tick organs, with higher prevalence of *R. parkeri* wild-type than *R. parkeri ompB^STOP^::tn* in salivary glands (1 day), midguts (1 and 6 days), and ovaries (3 days) (Fig. 6). These data indicate that OmpB is necessary for early rickettsial dissemination in the tick vector. At 12 h after capillary feeding, a significantly higher rickettsial load of *R. parkeri* wild-type than *R. parkeri ompB^STOP^::tn* was identified in midgut and ovary tissues. At 1 day after exposure, rapid clearance of *R. parkeri ompB^STOP^::tn* was observed in all tissues versus *R. parkeri* wild-type. At 3 and 6 days, there were no significant differences in rickettsial load between the strains in all tick tissues. No vertical transmission of *R. parkeri* wild-type or *R. parkeri ompB^STOP^::tn* was detected in *F*_1_ larvae (data not shown). These data indicate that OmpB is associated with rickettsial infection and dissemination in the tick vector. Although greater rickettsial loads were observed for *R. parkeri* wild-type after ingestion, replication kinetics did not vary between strains in the tick vector.

### *R. parkeri* wild-type-infected ticks cause more severe lesion in rats than *R. parkeri ompB^STOP^::tn*-infected ticks

After ticks were removed, samples from rats were collected, including skin at and distant from the bite site, heart, spleen, liver, and blood at 1, 3, and 6 days after infestation and when ticks were fully engorged to track the transmission of rickettsiae to the vertebrate hosts. The tissue samples were bisected and processed for gDNA extraction and subsequent qPCR. Only one skin sample from the bite site of ticks infected with *R. parkeri* wild-type at 6 days met the qPCR threshold for rickettsial count (6.95 × 10^1^ rickettsiae). Below the qPCR quantification threshold (>35 cycles), trace amounts of rickettsial DNA in rats exposed to ticks infected with *R. parkeri* wild-type were identified in the liver and heart at 3 days and blood at 6 days. For rats exposed to ticks infected with *R. parkeri ompB^STOP^::tn*, samples including blood at 3 days, 6 days, and fully engorged ticks and skin at the bite site at 3 days had trace amounts of rickettsial DNA. Sequence analyses of qPCR-positive rat tissue samples confirmed a 100% match with *R. parkeri* Portsmouth strain (GenBank accession number NC_017044.1).

**Fig 7:**
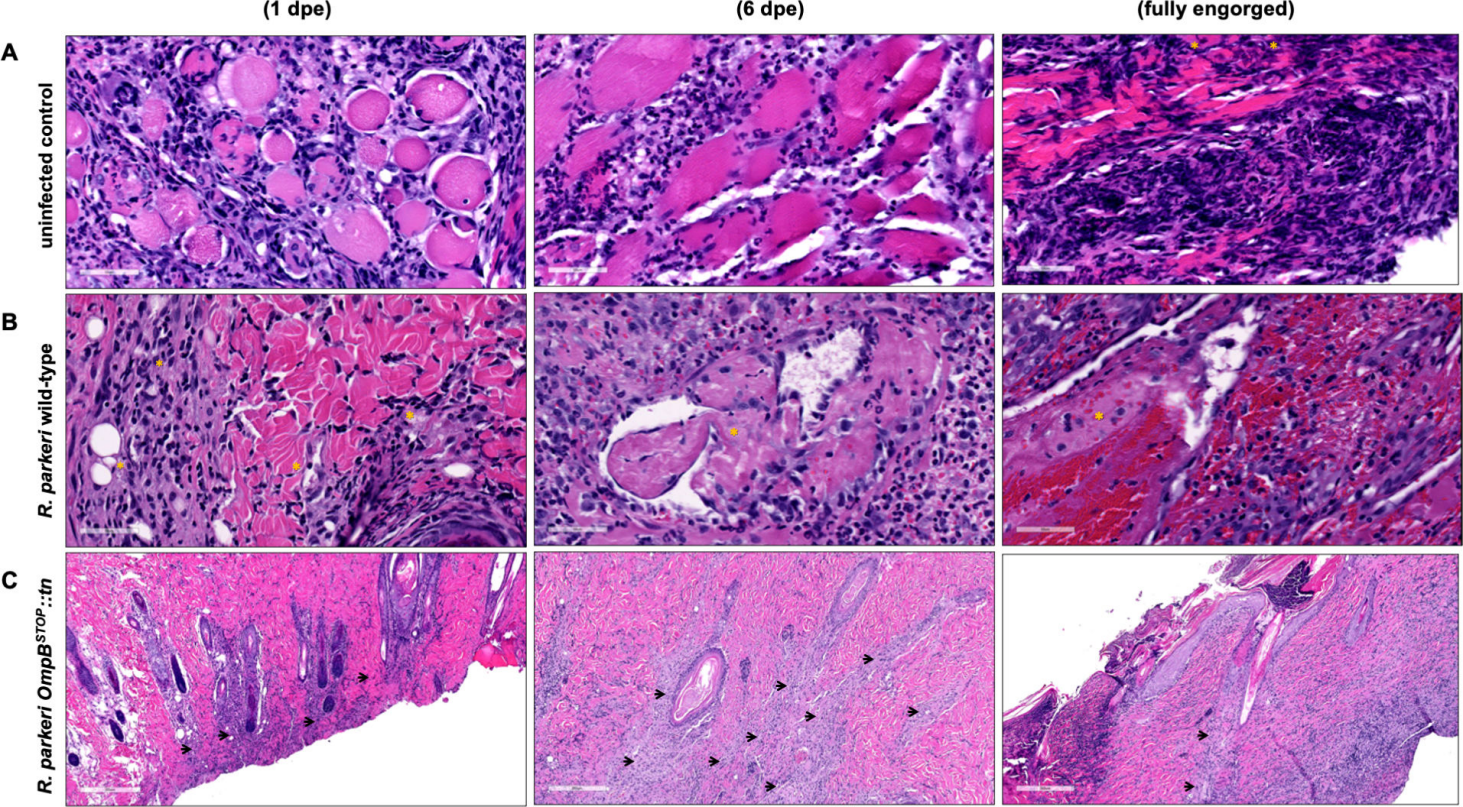
Histology of rat skin at tick infestation site at 1 and 6 days after exposure and after ticks were fed until fully engorged. Scale bars: *R. parkeri* wild-type at 1 day, 50 µm; all others, 1 mm. (**A**) Uninfected ticks (control). Panniculus carnosus muscle partially effaced by inflammation at all time points. (**B**) *R. parkeri* wild-type-infected ticks. Several subcutaneous and deep dermal vessels were partially occluded by fibrin thrombi and lined by hypertrophic endothelium among inflammation (indicated with asterisks). (**C**) *R. parkeri ompB^STOP^::tn*-infected ticks. Inflammatory exudate tracked into the overlying dermis along the deep dermal vasculature (indicated with arrows).

Rickettsial transmission to vertebrate hosts was analyzed by immunohistochemistry and hematoxylin and eosin (H&E) staining of the rat skin at and distant from the bite site, heart, spleen, and liver. Rickettsiae were not observed in immunohistochemistry sections. Independent of detectable rickettsiae at the tick attachment site, H&E staining revealed greater severity of lesions corresponding to the duration of tick feeding and the infection status of the tick (Table 1). There were no major differences in the histopathology scores of the heart, spleen, and liver of the rats exposed to both strains (data not shown). In the skin sample at the control (uninfected) tick infestation site, the dermis and subcutaneous adipose tissue were infiltrated by numerous neutrophils and macrophages and low numbers of lymphocytes, plasma cells, and eosinophils. The inflammation extended into the panniculus carnosus muscle, which had individual necrotic myofibers at 1 and 6 days after exposure (Fig. 7A). The skin from rats exposed to ticks infected with *R. parkeri* wild-type showed several deep dermal and subcutaneous vessels that were partially occluded by fibrin thrombi and lined by hypertrophic, inflamed endothelium. The inflammation included a mixture of neutrophils, macrophages, and reactive lymphocytes interspersed within plump (proliferating) fibroblasts and was most severe at the deeper levels of the skin and panniculus carnosus muscle (Fig. 7B). The sections from the rats exposed to ticks infected with *R. parkeri ompB^STOP^::tn* had changes that were most prominent in the subcutaneous tissue, including multifocal fibrin and macrophage infiltration. The inflammatory exudate tracked into the overlying dermis and the deep dermal vessels (Fig. 7C). There was less inflammation in the skin at the bite site of rats exposed to *R. parkeri ompB^STOP^::tn* than *R. parkeri* wild-type.

**TABLE 1:** Histology of tick infestation sites in *Rickettsia parkeri* wild type and *Rickettsia parkeri ompB^STOP^::tn*-infected rats^*c*^

	Histologic scoring^*a*^ at the indicated time point (day post-exposure)											
Time point Animal	Epidermal necrosis				Dermatitis				Panniculitis			
	1	3	6	Fully engorged	1	3	6	Fully engorged	1	3	6	Fully engorged
Control (uninfected ticks)	–	–	n/a	+	++^*b*^	–	+++^*b*^	++^*b*^	+++	+	+++^*b*^	n/a
*R. parkeri* wild-type-infected ticks (1)	+	++	+	+++^*b*^	++^*b*^	++^*b*^	++^*b*^	+++^*b*^	++	+++^*b*^	+++^*b*^	+++^*b*^
*R. parkeri* wild-type-infected ticks (2)	++	++	–	++	++^*b*^	++^*b*^	++^*b*^	++	n/a	+++^*b*^	+++^*b*^	+++^*b*^
*R. parkeri ompB^STOP^::tn*-infected ticks (1)	+	+	++	+++^*b*^	+	++	+	++^*b*^	n/a	++^*b*^	+++^*b*^	+++^*b*^
*R. parkeri ompB^STOP^::tn*-infected ticks (2)	+	++	–	+++	+^*b*^	++^*b*^	+	+++^*b*^	+^*b*^	+++^*b*^	++	+++^*b*^

^*a*^ Histologic scores for epidermal necrosis, dermatitis, or panniculitis: –, absent; +, mild change (finding rare or infrequent at high magnification); ++, moderate change (change observed in multiple high-power fields or large foci present); +++, marked change (change frequently observed in multiple high-power fields or severe in focal areas).

^*b*^ Diffuse inflammation present, affecting the superficial and deep aspect; absence of “superscript *b*” denotes inflammation restricted to the perivascular space.

^*c*^ Fully engorged: when ticks became fully engorged and dropped off the animal. The number in parentheses indicates the replicate of the hosts exposed to the indicated infected ticks. na, not available, i.e., missing or not represented in the section.

## DISCUSSION

Due to the obligate intracellular nature of *Rickettsia,* the steps of host cell attachment and invasion are critical for survival. Recent advances in transposon mutagenesis have facilitated studies of several rickettsial molecules in host infection (12, 22, 23, 25–31). The rickettsial protein OmpB is involved in adhesion and invasion in addition to impeding recognition by the host ubiquitylation and autophagy machinery in vertebrate cells (12, 32). The present results showed that mutational loss of functional OmpB in *R. parkeri* coincided with lower levels of rickettsial association and internalization in ISE6 cells *in vitro*, lower rickettsial loads in tick tissues *in vivo*, and lower levels of inflammation in rats bitten by *Rickettsia*-infected ticks. These results provide evidence that rickettsial OmpB functions in rickettsial association and internalization in ISE6 cells, infection in the tick vector, and pathogenicity in mammals.

Host cell association (22) and internalization (12, 23) assays were employed to assess the function of OmpB in tick and mammalian host cells. In ISE6 cells, the decreased ability of *R. parkeri ompB^STOP^::tn* to bind was coupled with reduced number of rickettsiae per host cell compared to *R. parkeri* wild-type. The association between OmpB and rickettsial internalization was most evident at 30–60 min after infection but was decreased by 90 min post-infection. Rickettsial internalization was influenced by OmpB but appeared to be mitigated over time. In contrast, cell binding and internalization of *R. parkeri ompB^STOP^::tn* in Vero cells were greater than *R. parkeri* wild-type. It has been demonstrated that *R. parkeri* OmpB is important for rickettsial infection of mammalian macrophages but, consistent with Vero cells used in the current study, not human dermal microvascular endothelial cells (12). The association and internalization experiments were performed with fewer Vero than ISE6 cells seeded to achieve similar confluence in culture, but the same MOI was used for experiments with both cell types. The observed differences between tick and mammalian cell binding and invasion appear to be influenced by several factors, including cell origin and rickettsial proteins other than OmpB. Unlike the homogenous Vero cell culture, tick-derived cell lines generated from embryonic tissues are heterogeneous (33–36), and the potential differences in individual cell-type interactions with rickettsiae are not known. In addition to OmpB, other rickettsial molecules including Sca0 (OmpA), Sca1, and Sca2 facilitate adherence and invasion of mammalian cells (9, 37–40). Consistent with the current observations, *Rickettsia felis* that were deficient in full-length Sca1 demonstrated decreased rickettsial attachment with no distinct internalization phenotype in ISE6 cells (22). Compensation for impaired Sca(s) function may be a mechanism for alternating between host cell backgrounds and requires further study.

Growth kinetic studies have been used previously to assess rickettsial virulence (12, 19, 22, 23, 27–29, 31, 41, 42). In the ISE6 cells, no differences in replication were observed between *R. parkeri* wild-type and *R. parkeri ompB^STOP^::tn*, similar to previous findings for *R. parkeri* lacking functional RickA and Sca2 (19). However, disruption of Sca1 in *R. felis* resulted in increased rickettsial growth in ISE6 cells (22). In contrast with the results in ISE6 cells, *R. parkeri ompB^STOP^::tn* showed increased numbers of rickettsiae in Vero cells at 96 h. In Vero cells, IFA showed that infection by *R. parkeri ompB^STOP^::tn* was heavy, with the monolayer intact through 96 h, in contrast with focal infection and rapid cellular detachment observed with *R. parkeri* wild-type after 72 h. These differences in rickettsial growth and disruption in the monolayer suggest that OmpB-deficient rickettsiae can disseminate to neighboring cells at 96 h, while exhaustion of host cell resources and adjoining cells necessary to accommodate sustained rickettsial replication may have been a limit for *R. parkeri* wild-type. Although host cell factors may contribute to *R. parkeri* growth in mammalian cells, future studies are warranted using comprehensive rickettsial transcript analyses to characterize mutant infection kinetics and molecular compensation when an individual molecule is disrupted in varied host cells.

Ticks acquire rickettsiae during bloodmeal acquisition. Alternatively, they may inherit the infection as rickettsiae are passed from females to progeny. Rickettsiae that are ingested by female ticks enter the tick gut and disseminate to salivary glands for horizontal transmission or ovaries for vertical transmission (43, 44). In the present study, tick infection and transmission bioassays with *Rickettsia*-free *A. maculatum* (19) and rats enabled interrupted feeding and capillary inoculation into ticks on mammals that are exposed naturally to *R. parkeri* (45). Quantification in tick tissues by qPCR showed that rickettsial load of *R. parkeri ompB^STOP^::tn* was lower than *R. parkeri* wild-type in all tissues at 1 day after exposure, suggesting that OmpB may facilitate early rickettsial infection and dissemination in the tick. The amount of rickettsiae subsequently decreased in ticks exposed to *R. parkeri* wild-type or *R. parkeri ompB^STOP^::tn*, suggesting that orally acquired rickettsiae result in diminishing rickettsial load. The observed *R. parkeri ompB^STOP^::tn* dissemination to all tick organs and limited replication are consistent with observations in *R. parkeri* lacking functional RickA and Sca2 (19). The mechanisms of rickettsial dissemination in ticks are not known but may involve tracheal epithelial cells, hemolymph cells, and cell-free rickettsiae in the hemolymph (19, 43, 46–49). Furthermore, it is unknown whether rickettsial dissemination in ticks is an active, driven by the bacteria, or passive process. Although *R. parkeri* is vertically transmitted by *A. maculatum* (50, 51), neither *R. parkeri* wild-type nor *R. parkeri ompB^STOP^::tn* mutant was detected in progeny from exposed ticks despite rickettsial detection in ovarian tissue. Infection of adult ticks using a method distinct from natural acquisition has provided mixed results (19, 52–54) and typically does not reflect the stable transmission observed in ticks that are constitutively infected in all life cycle stages (46, 51, 55). Further study is required to evaluate the temporal aspects of reproductive tissue infection that may affect vertical transmission of rickettsiae.

Previous analysis of *R. parkeri* infection in *A. maculatum* salivary glands showed an association between rickettsial load, transmission kinetics, and lesion formation at the tick attachment site (45). Although rickettsial DNA from *R. parkeri* wild-type and *R. parkeri ompB^STOP^::tn* were detected in vertebrate hosts, only *R. parkeri* wild-type was quantifiable at 6 days after exposure by qPCR. In addition, immunohistochemistry staining did not identify rickettsiae in skin samples at the tick attachment site. Discrepancies between molecular and microscopic analyses relative to detection of rickettsiae delivered by ticks may be due to assay sensitivity or an artifact of sampling (56, 57). In the current study, independent of detectable rickettsiae at the tick attachment site, hematoxylin and eosin staining showed greater severity of lesions corresponding to the duration of tick feeding and the infection status of the tick. However, we observed that host inflammation was lower when exposed to ticks infected with *R. parkeri ompB^STOP^::tn* versus *R. parkeri* wild-type. Therefore, a lower rickettsial load of *R. parkeri ompB^STOP^::tn* in the tick salivary glands was consistent with reduced transmission of rickettsiae and less severe lesions at the feeding site, evidence in support of the function of OmpB in transmission and pathogenicity.

Limitations of the present study included the use of only two rats for each rickettsial strain and time point in the bioassays and histologic evaluation, especially considering the varied range of results (Table 1). Although these experiments were performed as independent replicates, with consideration of the use of vertebrate hosts and parasite load, a larger study with individual tick/host pairings may be necessary for confirmation of the results.

In conclusion, the present results showed that rickettsial OmpB is associated with rickettsial attachment and internalization in tick cells and infectivity in ticks and mammals. During tick acquisition of blood, transcription of *ompB* was detected in *Rickettsia*-infected ticks, suggesting that OmpB may be important for rickettsial transmission by the vector (20, 21, 58). Although at reduced loads in tick tissues, the lack of functional OmpB did not prevent dissemination of rickettsiae when acquired during feeding. As the specific avenue of rickettsial dissemination in the tick is not known, future studies are required to examine the *R. parkeri* and tick hemocyte interaction and the role of OmpB compared to what is known for mammalian macrophages (12). Furthermore, examination of the autophagic response to *R. parkeri* infection with or without OmpB in tick hemocytes is necessary as hemocytes are considered mediators of the tick immune response to pathogens (59). Rickettsiae may use other rickettsial factors in tick cells such as OmpA (39), RickA, Sca2 (27, 60), and Sca4 (28) to compensate for the loss of OmpB, and further studies are needed to characterize the effects of protein deletion on the composition of rickettsial surface antigens.

## MATERIALS AND METHODS

### Cell culture

*Ixodes scapularis* embryonic-derived ISE6 cells (RRID: CVCL_Z170, ATCC) and African green monkey kidney epithelial (vertebrate-derived) Vero cells (CCL-81, RRID: CVCL_0059, ATCC) were maintained as described (61, 62). Briefly, ISE6 cells were cultured with Leibovitz L-15B medium (Gibco-BRL, 41300039, NY) supplemented with 10% heat-inactivated fetal bovine serum (FBS; HyClone, AG29689170, UT) and 10% tryptose phosphate broth (pH 6.8–7.0) (Sigma-Aldrich, MO) and maintained at 32°C in a 5% CO_2_ humidified incubator. Vero cells were maintained in Dulbecco Modified Eagle Medium (Gibco, AG29689170, NY) supplemented with 5% heat-inactivated FBS and incubated at 34°C in a 5% CO_2_ humidified incubator. Rickettsial infection was performed at 34°C in a 5% CO_2_ humidified incubator.

### *Rickettsia* strains, propagation, and purification

*R. parkeri* wild-type (Portsmouth strain) and mutant *R. parkeri ompB^STOP^::tn* (derived from the Portsmouth strain) were propagated in Vero cells as described previously (12, 63). Rickettsiae were counted as described previously (24). The infected cells were lysed with a 27-gauge needle, rickettsiae were separated from the cell debris by centrifugation (275 × *g*) for 3 min at 4°C, and the supernatant was filtered through a 2-µm syringe filter (Whatman-Cytiva, MA). Rickettsiae were stained with a Bacterial Viability Kit (Live/Dead BacLight; Invitrogen, CA) for 15 min and counted with a counting chamber (Petroff-Hausser; Hausser Scientific, PA) under a fluorescent microscope.

### *R. parkeri ompB^STOP^::tn* clonal confirmation

ISE6 cells were seeded on 24-well plates at 4 × 10^5^ cells per well and incubated at 32°C for 48 h before infection. *R. parkeri* wild-type (passage 3) and *R. parkeri ompB^STOP^::tn* (passage 3) were used in separate infection bioassays. Infected cells were collected for PCR and Western blot at 3 days after infection. To confirm clonality of OmpB mutant by PCR, two sets of primers were designed: primer set 1 to amplify gDNA of upstream OmpB using RpompB129FJJ and RpompB224RJJ primers and primer set 2 downstream over the transposon insertion using RpompB462Fw and RpompB741Rv primers. Two sets of PCR primers were designed from the reference sequence of *R. parkeri* (Portsmouth strain) (GenBank accession number NC_017044.1, National Center for Biotechnology Information, MD) capturing the insertion into *ompB* by the primer set 1 (Table 2; Fig. S1) (18, 44, 55, 63–66). Band size of 300 bp in an agarose gel indicated *R. parkeri* wild-type, and 3,300 bp indicated *R. parkeri ompB^STOP^::tn* clonality (Fig. S1). The thermocycler conditions for PCR were 95°C for 3 min, 34 cycles at 95°C for 30 s, 55°C for 30 s, 72°C for 30 s, and 72°C for 7 min.

**TABLE 2:** Primers and probes used for quantitative polymerase chain reaction to detect *Rickettsia* species and host cells^*a*^

Primer set or probe	Sequence (5′−3′)	Partial gene amplified	Reference or source
RpompB129FJJ	CAAATGTTGCAGTTCCTCTAAATG		(56)
RpompB224RJJ	AAAACAAACCGTTAAAACTACCG		
RpompB462Fw	GGCAGTAGTACCGTCTGTACC	*R. parkeri ompB* (primer set 1)	Present study
RpompB741Rv	GCTGCTACCGACTTTAACGTT	*R. parkeri ompB* (primer set 2)	Present study
RaRp17.181F RaRp17.289 Rp17kDa	GCATTACTTGGAGCAGTTCT CGCCATTCTACGTTACTACC FAM-TGCAGAGCTTACCTCACAGAGAGCTT	*R. parkeri* 17-kDa surface antigen	(66)
AmacMIF.18F AmacMIF.99R AmacMIF.63	CCAGGGCCTTCTCGATGT CCATGCGCAATTGCAAACC HEX–TGTTCTCCTTTGGACTCAGGCAGC–BHQ-1	*A. maculatum mif*	(67)
Ratcfd121F RatcfdRev RatcfdHex	GCTTCAGTGCAAGTGAATGG TGCCACTCACACTCCATCC HEX–TGGATGAGCAGTGGGTGCTGA–BHQ-1	Rat *cfd* (complement factor D)	(68) (45)
Vero b-actin.61F Vero b-actin.170 R Vero b-actin.116	TGAAGTGTGACGTGGACATCCATA GGCAGTAATCTCCTTCTGCATCCT TGGCACCACCATGTACCCTGGCATTGCT	Vero β-actin	(69)
ISE6_calFW ISE6_calREV ISE6_cal.67	AGCAGGGAACTTTCAAGCTG AGAAAGGCTCGAACTTGGTG HEX-AGACCTCTGAAGATGCCCGCTTT	*I. scapularis* calreticulin (*crt*)	(19)

^*a*^ BHQ-1, Black Hole Quencher; FAM, 6-carboxyfluorescein; HEX, hexachlorofluorescein.

The bicinchoninic acid assay was performed to measure protein concentration for Western blot. Protein (80 µg) from *Rickettsia*-infected ISE6 cells was loaded on precast gels (Mini-PROTEAN TGX, Bio-Rad, CA), transferred onto a nitrocellulose membrane (pore size, 0.45 µm) (Bio-Rad, Germany), and blocked with 5% skim milk (Bio-Rad, CA) for 1 h. Membranes were incubated with primary anti-*Rickettsia* (Rc_PFA_) polyclonal antibodies (64) at 1:1,000, anti-OmpB (amino acids of OmpB 36–218) at 1:1,000 (12), and anti-β-actin at 1:1,000 as internal load control (Sigma-Aldrich, Israel) in 5% skim milk. The secondary antibodies were anti-rabbit IgG and anti-mouse IgG conjugated with horseradish peroxidase (Sigma-Aldrich, USA) at 1:10,000. Protein signals were developed by Western blot substrate (SuperSignal West Femto, ThermoFisher Scientific, IL), and the signal was captured by a fluorescence imager (ChemiDoc, Bio-Rad) (Fig. S1).

### Cell association and internalization assays in tick cells

Cell association was determined with qPCR, and internalization was evaluated with IFA. ISE6 cells were seeded on 24-well plates at 4 × 10^5^ cells per well and incubated at 32°C 48 h before infection, and Vero cells were seeded on 24-well plates at 8 × 10^4^ cells per well and incubated at 34°C for 24 h before infection; cells were plated without coverslips for the cell association and with 12-mm coverslips for the internalization assays (Chemglass Life Sciences, Germany). Purified cell-free rickettsiae of *R. parkeri* wild-type or *R. parkeri ompB^STOP^::tn* were added to tick cell cultures at MOI 5. Cells and coverslips were collected at 0, 5, 10, 15, 20, 30, 60, and 90 min after infection. Supernatant was removed, and cells were washed with phosphate-buffered saline (1× PBS) with 0.5% Tween. Portions of rickettsial and host genes were amplified from extracted gDNA (DNeasy Blood and Tissue Kit, Qiagen, MD) by qPCR, with minor modifications as described previously (19). Briefly, the 17-kDa surface antigen for *R. parkeri,* calreticulin for ISE6 cells, and β-actin for Vero cells (Table 2) were quantified using standard curves by qPCR (LightCycler 480, Roche, IN). The thermocycler conditions were 95°C for 3 min, 45 cycles at 95°C for 15 s, 60°C for 1 min, and 40°C for 40 s. Subsequently, the quantity of rickettsiae was normalized relative to the host cell.

For the internalization assay, coverslips were washed with 1× PBS and fixed with 4% paraformaldehyde and 4% sucrose (4% PFA) for 10 min. Coverslips were washed three times with 1× PBS T as described previously (12). Coverslips were incubated in a humidified chamber throughout staining. Samples were stained with Rc_PFA_ (1:1,000) in 2% bovine serum albumin (BSA, Sigma-Aldrich, USA) without permeabilization for 1 h, washed three times with 1× PBS T, and incubated with goat anti-rabbit Alexa 594 (1:500) (Invitrogen, MA) in 2% BSA to assess extracellular rickettsiae. Cells were permeabilized with 0.5% TritonX-100 (Fisher Scientific, NH) in PBS for 15 min and washed three times with 1× PBS T. Samples were blocked with 3% BSA in 1× PBS for 1 h. Intracellular rickettsiae were visualized after labeling with Rc_PFA_ (1:1,000) in 2% BSA for 1 h, washing three times with 1× PBS T, and incubating with goat anti-rabbit Alexa 488 (1:500) (Invitrogen) in 2% BSA for 1 h. Rickettsiae were imaged with a confocal microscope (Nikon A1, Nikon, NY). Bright field images were also captured. No primary antibody was used as a control for specificity of antibody.

### Growth kinetics analysis in ISE6 and Vero cells

Rickettsiae were semi-purified and counted to assess *in vitro* growth kinetics as described previously (65). ISE6 cells were seeded at 4 × 10^5^ cells per well and incubated for 48 h at 32°C before infection. Vero cells were seeded on 24-well plates with and without coverslips at 8 × 10^4^ cells per well and incubated at 34°C. *R. parkeri* wild-type or *R. parkeri ompB^STOP^::tn* was added to ISE6 and Vero cells (MOI 1) with plate centrifugation (500 × *g*) for 5 min at 4°C to facilitate rickettsial attachment to host cells. ISE6 cells were incubated at 32°C and Vero cells at 34°C, each for 1 h. Cells were washed with 1× PBS and collected for the initial count. Infected cells were incubated, and samples were collected at 24, 48, 72, and 96 h for gDNA extraction and quantification of rickettsial loads by qPCR for 17-kDa surface antigen (66). Quantification of the 17-kDa surface antigen was performed by referencing it against the standard curves to get the number of rickettsiae. At the same time points, infected and uninfected cells on coverslips were fixed with 4% PFA and processed for IFA. ISE6 or Vero cells were seeded on 12-mm coverslips in 24-well plates. After rickettsial infection, coverslips for growth kinetics were washed with 1× PBS and fixed with 4% PFA for 10 min, followed by washing three times with 1× PBS T as described previously (19). Coverslips were permeabilized with 0.5% TritonX-100 in PBS for 15 min and washed three times with 1× PBS T. Samples were blocked with 3% BSA in 1× PBS for 1 h, incubated with anti-*Rickettsia* (Rc_PFA_, 1:1,000) in 2% BSA for 1 h, washed three times with 1× PBS T, probed with goat anti-rabbit Alexa 488, Alexa 594 phalloidin, and 4′,6-diamidino-2-phenylindole (1:500 in 2% BSA), and imaged with a confocal microscope (10 fields) along with a bright field microscope.

### Tick infection, dissemination, and transmission bioassays

*Rickettsia*-free *A. maculatum* were originally received from the Centers for Disease Control and Prevention for distribution by BEI Resources, NIAID, NIH (adult *A. maculatum*, NR-44382). Sprague-Dawley rats (age, 5–6 weeks old) were used for experiments. Ticks (5 males and 10 females) were prefed in a modified 50-mL conical tube, which was placed on the back of each of four rats per biological replicate, for tick infection, dissemination, and transmission (Fig. S2). At 3 days after attachment, female ticks were removed from the host and exposed to *R. parkeri* wild-type or *R. parkeri omp^STOP^::tn* by capillary feeding as described previously (50, 52). *R. parkeri* wild-type or *R. parkeri ompB^STOP^::tn* (5 × 10^7^ rickettsiae) or uninfected media control was mixed with 0.85% sodium chloride (weight in volume) and 0.1% rhodamine B (weight in volume) and offered to naïve *A. maculatum* ticks via a capillary placed over the hypostome at 37°C in a humidified incubator, with rhodamine B serving as a marker for rickettsial consumption by immobilized ticks after 12 h (19).

Rhodamine B-positive ticks (five females) were returned to each rat host within 1 h after the 12-h capillary feeding event. A portion of the ticks were immediately assessed for rickettsial dissemination at 12 h after exposure by dissecting five rhodamine B-positive ticks to recover salivary glands, midguts, and ovaries. The remaining rhodamine B-positive ticks that were returned to the host to continue feeding were removed from the rat after 1, 3, and 6 days and when fully engorged. Tick tissues were recovered immediately after removal from the host. At the time of tick removal, blood and selected rat tissues, including skin (diameter, 0.5 cm) at and away from the bite site, heart, spleen, and liver, were collected and divided into two equal parts for qPCR and histopathology. Tick and rat tissues were processed for gDNA extraction, and rickettsial load was determined by qPCR (65). Briefly, tick samples and portions of rat skin and other tissue samples were homogenized (TissueLyser II, Qiagen, MD) using 3.2-mm stainless steel beads (McMaster-Carr, GA) at 30 m/s for 1.5 min. The gDNA was extracted (DNeasy Blood and Tissue Kit, Qiagen) and subjected to qPCR. For tick samples, quantification of the 17-kDa surface antigen was performed by referencing it to a standard curve to get the total number of rickettsiae. Subsequently, the number of rickettsiae was normalized relative to the tick host (10^5^ of tick cells). For rat samples, *cfd* and *R. parkeri* 17-kDa surface antigen (Table 2) were amplified. Positive qPCR results for rat tissue samples were confirmed by amplifying the 17-kDa surface antigen gene with primers RaRp17.181F and RaRp17.289 by qPCR and thermocycler conditions as described previously (56, 66). Amplicons were visualized by electrophoresis on a 1% agarose gel, and the PCR products were extracted from the agarose gel (Wizard SV Gel and PCR Clean-Up System, Promega, WI). The amplicons were then cloned into pCR4-TOPO vector (Thermofisher), and Sanger sequencing was performed. Vertical transmission was assessed by allowing four fully engorged ticks to lay F_1_ eggs, collecting the F_1_ larvae for gDNA extraction, and evaluating rickettsial infection by qPCR to the 17-kDa surface antigen and tick macrophage migration inhibitory factor (*mif*) genes (19, 66).

### Histopathology and immunohistochemistry

Rat tissue samples were fixed with 10% neutral buffered formalin, embedded in paraffin, and sectioned (thickness, 4 µm) for hematoxylin and eosin staining (VWR International, PA). Immunohistochemistry was performed for rickettsiae by staining serial sections with a rabbit anti-Rc_PFA_ polyclonal antibody as described previously (56). Tissue sections were examined by a board-certified veterinary anatomic pathologist in a randomized and blinded manner.

### Statistical analysis

Data analyses were performed with statistical software (Prism 9.0, GraphPad, CA). Numerical data were analyzed using two-way analysis of variance with Šidák multiple comparison test unless stated otherwise. Statistical analyses of tick samples were processed using two-way ANOVA for rickettsial loads and Fisher exact test for rickettsial prevalence. Statistical significance was defined by *P* ≤ 0.05.

## SUPPLEMENTAL MATERIAL

Supplemental figures iai.00515-23-s0001.docx Figures S1 and S2.

ASM does not own the copyrights to Supplemental Material that may be linked to, or accessed through, an article. The authors have granted ASM a non-exclusive, world-wide license to publish the Supplemental Material files. Please contact the corresponding author directly for reuse.
