# Supplementary material for: Critical roles of Rickettsia parkeri outer membrane protein B (OmpB) in the tick host
Source: Infect Immun. 2024 Jan 11;92(2):e00515-23. doi: 10.1128/iai.00515-23 (PMC10863407; doi:10.1128/iai.00515-23)
Supplement: Supplemental figures — Figures S1 and S2. [file iai.00515-23-s0001.docx]

**Supplementary Figure 1**

**
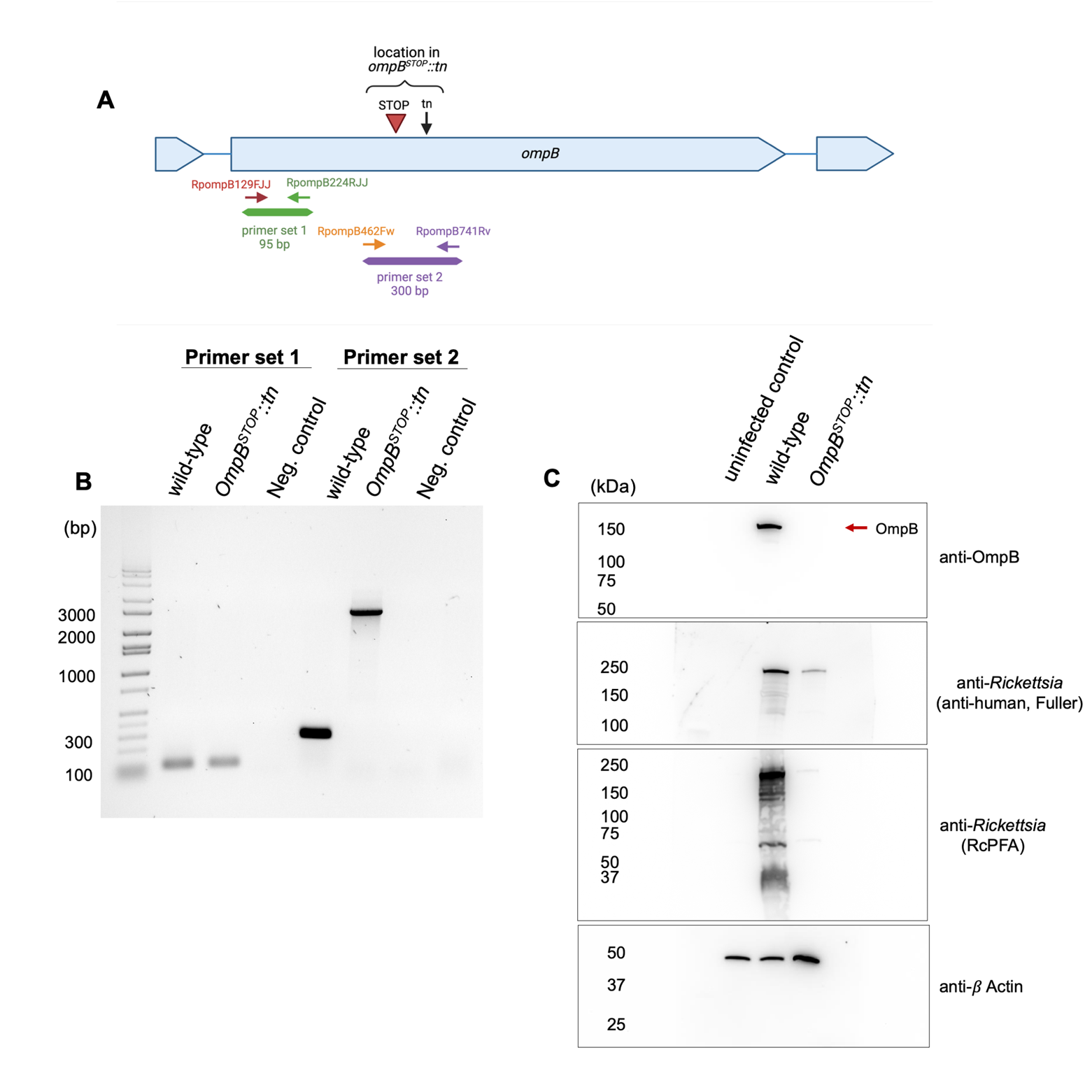
**

**FIG S1.** Clonality of *R. parkeri* wild-type and *R. parkeri* *ompB^STOP^::tn*. (A) Schematic showing transposon insertion (leading to early STOP codon) and 2 primer sets that were designed. Primer set number 2 captured the transposon insertion. Created with BioRender.com. (B) Agarose gel electrophoresis showing clonality of *R. parkeri* wild-type and *R. parkeri* *ompB^STOP^::tn* after amplifying with PCR. (C) Western blot of uninfected, *R. parkeri* wild-type-infected, and *R. parkeri ompB^STOP^::tn*-infected ISE6 cells that were detected and developed with 3 different conditions including anti-*Rickettsia*, anti-OmpB (12), and anti-β actin. Although less *R. parkeri ompB^STOP^::tn* than *R. parkeri* wild-type was present in the Western blot, evidenced by anti-*Rickettsia* staining, the suppression of *ompB* in *R. parkeri ompB^STOP^::tn* was confirmed by the absence of an OmpB band on the Western blot and DNA sequencing.

**Supplementary Figure 2**

**
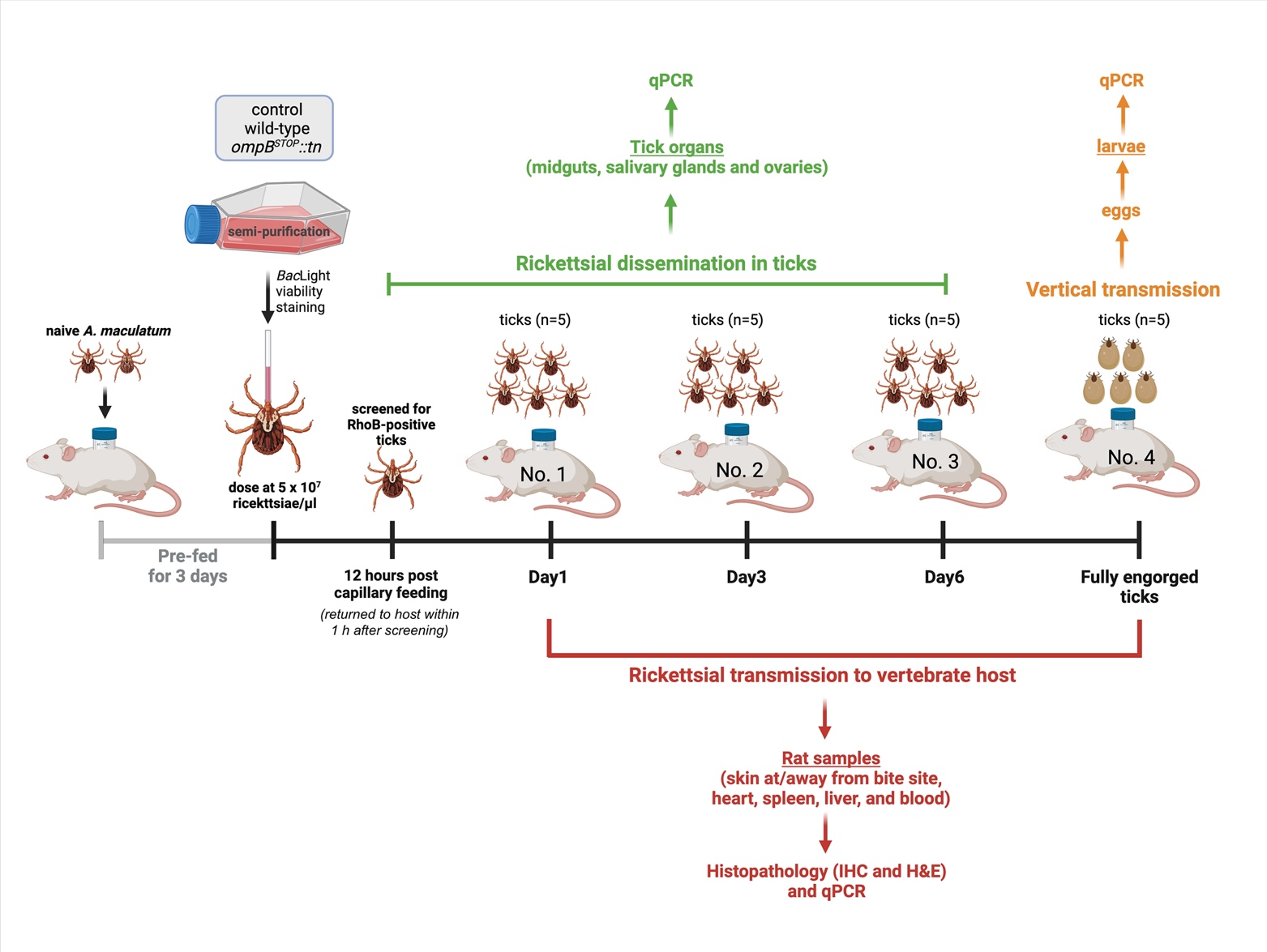
**

**FIG S2.** *In vivo* experimental design to study *R. parkeri* OmpB in *A. maculatum* tick infection and transmission in the vertebrate host. Naïve ticks were prefed for 3 days on rats. Purified rickettsiae were mixed with 0.1% rhodamine B and 0.85% sodium chloride for capillary feeding. At 12 h after capillary feeding, rhodamine B-positive ticks were returned to vertebrate hosts within 1 h after screening. Tick and rat samples were collected and processed at the times shown. ***Abbreviations:*** H&E, hematoxylin and eosin; IHC, immunohistochemistry. Created with BioRender.com.
